# In vitro the behaviors of metastasis with suppression of VEGF in human bone metastatic LNCaP-derivative C4-2B prostate cancer cell line

**DOI:** 10.1186/1756-9966-31-40

**Published:** 2012-05-01

**Authors:** Lei Yang, Shuo You, Vikas Kumar, Chaoyue Zhang, Ya Cao

**Affiliations:** 1 Department of Orthopedics, the Third Xiangya Hospital of Central South University, Changsha, Hunan, 410013, China; 2 Department of Endocrinology, the Second Xiangya Hospital of Central South University, Changsha, Hunan, 410011, China; 3 School of Medicine, University of Pittsburgh, Pittsburgh, PA, 15261, USA; 4 Cancer Research Institute of Xiangya School of Medicine, Central South University, Changsha, Hunan, 410078, China

**Keywords:** Metastasis, Bone, Prostate cancer, VEGF

## Abstract

**Background:**

Vascular endothelial growth factor (VEGF) is a signal protein produced by cells that stimulates vasculogenesis and angiogenesis. VEGF is believed to implicate poor prognosis in various cancers. The overexpression of VEGF may be an early step in the process of metastasis.

**Methods:**

ELISA was used to investigate the levels of VEGF, bFGF and IL8 in human bone metastatic LNCaP-derivative C4-2B prostate cancer cell line and its parental cell line, LNCaP and to determine the effect of bevacizumab on reducing the level of VEGF. Cell proliferation assay, invasion assay and in vitro angiogenesis assay were performed under the condition with bevacizumab or control IgG.

**Results:**

Human bone metastatic LNCaP-derivative C4-2B prostate cancer cell line expressed a higher level of VEGF than its parental primary prostate cancer cell line LNCaP. The effect of bevacizumab is dose-dependent and time-dependent: 100 μg/mL of bevacizumab and 3-day treatment was more effective than low-dose and lesser-day treatment for decreasing the level of VEGF. Bevacizumab is able to suppress cell proliferation, angiogenesis and invasion in human bone metastatic C4-2B prostatic cancer cell line.

**Conclusions:**

The overexpression of VEGF can be inhibited by bevacizumab in human bone metastatic cancer cell line. The behaviors of metastasis involving proliferation, angiogenesis and invasion are suppressed by anti-VEGF therapy.

## Background

Most of the time, when patients have cancer in their bones, it is caused by metastatic cancer, or cancer that has spread from elsewhere in the body to the bones. It is much less common to have a primary bone cancer that arises from cells that make up the bone. Surgery, chemotherapy and radiation therapy are the three main types of treatment for bone cancer. Unfortunately, there are risks and side effects associated with each of the treatments for bone cancer. The main risks associated with surgery include infection, recurrence of the cancer, and injury to the surrounding tissues that may cause loss of sensation, strength or function, or even cause amputation. The medications of chemotherapy are designed to kill rapidly dividing or growing cells, but unfortunately normal cells are also adversely affected. Radiation therapy damages the surrounding skin and soft tissue and impairs wound healing. There has been much recent advancement in the understanding and treatment of bone cancer. This has led to more focused radiation therapy to reduce the risk to surrounding tissues, less side effects, and improved treatment options, including limb-salvaging surgery, that decrease the need for amputation. There is currently much work being conducted in each of these areas as well as investigations into the mechanisms of development of metastatic cancer. It is hoped that a better understanding of specific causes and mechanisms of metastatic cancer will lead to advanced therapy that targets specific metastatic cancer cells with limited risk to other normal cells.

Tumors are able to grow independently of vascularization until they reach a size of approximately 2 mm. At this size the tumor is unable to grow further due to the lack of nutrients and gas exchange, resulting in tumor dormancy [1]. Continued growth requires tumor vascularization. Cancer cells are able to induce angiogenesis by secreting angiogenic factors including vascular endothelial growth factor (VEGF) in order to activate certain actions by endothelial cells [2]. Normally, endothelial cells divide infrequently, being held in check by angiogenesis inhibitors. Once activated the endothelial cells secrete matrix-metalloproteases which begin to digest the extracellular matrix surrounding the blood vessels. The endothelial cells can then remodel the tissue. These migrating cells also divide and increase in number, eventually organizing into discrete tubules. Eventually these tubules connect via anastomosis to form the neovasculature of the tumor. The up-regulated VEGF promotes the activation of matrix-metalloproteases [3-5]. We hypothesize that an anti-VEGF agent is able to maintain tumor dormancy, and we aim to prove this hypothesis using in vitro cell growth assay, angiogenesis assay and invasion assay. For solid tumors, such as prostate cancer, breast cancer and lung cancer, there is the chance that the cancer will become advanced and spread to the bone. In fact, for prostate cancer the bone is the most common site of recurrence: approximately 80% of prostate cancer recurrences are in the bone [6]. In this study, we will report how anti-VEGF therapy affects the growth and invasion of the bone metastatic prostate cancer cell.

## Materials and methods

### Cell culture and reagents

Human bone metastatic prostate cancer C4-2B cell line is a derivative of the LNCaP prostate cancer cell line with androgen-independent characteristics. C4-2B cells were obtained from ViroMed Laboratories, and LNCaP cells were purchased from American Type Culture Collection (Manassas, VA). Both C4-2B and LNCaP cells were maintained as monolayer cultures in RPMI 1640 medium supplemented with 2 mM L-glutamine, 10% fetal bovine serum and penicillin-streptomycin in a humidified atmosphere of 5% CO_2_ at 37°C. Human microvessel cells (VEC Technologies company, Rensselaer, New York) were cultured in endothelial cell growth medium (PromoCell, Heidelberg, Germany) in a humidified atmosphere of 5% CO_2_ at 37°C.

Bevacizumab (Genentech, San Francisco, CA) is a recombinant humanized monoclonal IgG1 antibody that contains human framework regions and the complementarity-determining regions of a murine antibody that binds to and inactivates all isoforms of VEGF.

### VEGF, bFGF and IL-8 ELISA assays

The secretion of VEGF, basic fibroblast growth factor (bFGF) and interleukin 8 (IL-8) by C4-2B cells to culture medium was quantified by an enzyme-linked immunosorbent assay (ELISA). A density of 2 × 10^5^ of C4-2B, LNCaP and human microvessel cells were separately plated in a six-well plate. Human bone metastatic prostate cancer C4-2B cells were also co-cultured with human microvessel cells. All cultures were performed in triplicate. When the cells reached 90% confluence on the third day after they were seeded, the media were changed to complete culture media with 25 or 250 μg/mL bevacizumab, or an equal amount of IgG1. The cell culture media were collected at 72 hours after treatment in culture medium with 2% FBS in 5% CO_2_ at 37°C. The levels of VEGF, bFGF and IL-8 in the supernatants were measured with an ELISA kit (Quantikine; R&D Systems, Minneapolis,MN) according to the manufacturer’s instructions.

### Cell proliferation assay

A density of 5×10^3^ cells per well was seeded on 96-well-plate overnight in complete culture medium and then treated with bevacizumab or control IgG or recombinant human VEGF in complete culture medium without fetal bovine serum for a 3-day incubation. The cell numbers were measured every 24 hours by mitochondrial 3-(4,5-dimethylthiazol-2-yl)-5-(3-carboxymethoxyphenyl)-2-(4-sulfophenyl)-2 H-tetrazolium, inner salt (MTS) assay with use of the CellTiter 96 Aqueous One Solution Cell Proliferation Assay (Promega) according to the manufacturer’s instructions.

### Invasion assay

When C4-2B cells reached below 80% confluence, serum containing medium was removed and replaced with serum-free medium containing bevacizumab (100 μg/mL) or an equal amount of IgG, and cultures were returned to an incubator for 24 hours. The in vitro invasion assay was performed with a 24-well collagen-based cell invasion assay kit (Millipore). 2 × 10^5^ of C4-2B cells in 300 μl culture medium containing 100 μg/ml bevacizumab or IgG were placed into an invasion chamber consisting of a 24-well collagen-based plate. In order to observe the direct role of VEGF on the invasion of C4-2B cells, recombinant human VEGF (100 ng/ml) was added to the lower chamber. The cells were incubated for 24 h at 37°C in a 5% CO_2_ incubator. The non-invading cells in the media were discarded from the top of the insert. The invasive cells on the lower surface of the membrane were stained by the green fluorescent dye Calcein AM (Invitrogen) in PBS at 37°C for 1 h. The fluorescently labeled cells were photographed under a fluorescence microscope. The fluorescence of the invaded cells was read by a microplate reader at excitation/emission wavelength of 530/590 nm.

### In vitro angiogenesis assay

When C4-2B cells reach 80% confluence, they were cultured in serum-free RPMI1640 treated with bevacizumab (100 μg/mL) or an equal amount of IgG for 24 h. The conditioned media were collected, centrifuged, and transferred to fresh tubes. Human microvessel cells were maintained in complete media and starved for 4 h, were trypsinized and seeded in each well with conditioned culture medium from C4-2B with 100 μg/mL bevacizumab or an equal amount of IgG1 on growth factor-containing ECMatrixgel (Millipore, Billerica, MA) in a 24-well-plate, incubated in 5% CO_2_ at 37°C for 10 hours, and inspected for tube formation with an inverted light microscope (Olympus, Tokyo, Japan). Three random fields per well were examined at 40× magnification, and the values were averaged. The pattern/value association criteria for tube formation are: 0, individual cells, well separated; 1, cells beginning to migrate and align themselves; 2, capillary tubes visible without sprouting; 3, sprouting of new capillary tubes; 4, closed polygons beginning to form; and 5, complex meshlikestructures developing. Each well was photographed using an inverted microscope with a digital camera. The images were taken at 10× magnification and the total lengths of the tubes were measured with Image J (Image Processing Analysis in Java, ver. 1.42; developed by Wayne Rasband, National Institutes of Health, Bethesda, MD; available at http://rsb.info.nih.gov/ij/index.html).

### Statistical analysis

Comparison between the two groups was performed using the student’s *t*-test. A P value of less than 0.05 was considered significant and a P value of less than 0.01 was considered highly significant. Microsoft® Office Excel 2003 SP3 was used for data analysis.

## Results

### Expression of VEGF, bFGF and IL-8

To screen for the expression of angiogenic factors in prostate cancer cell and its bone metastatic cell, three angiogenic factors in conditioned media were detected with ELISA. The secreted VEGF by the parental LNCap cell line and its derived bone metastatic cell line C4-2B was detected. The production of bone metastastic cell line C4-2B (294.47 ± 31.99 pg/ml) was significantly higher than its parental cell line LNCap (204.40 ± 23.32 pg/ml, P = 0.016). The secreted bFGF and IL-8 protein were not detected in bone metastatic cell line C4-2B and its paretental LNCap cell line by EILSA.

### Bevacizumab suppressed VEGF from C4-2B and microvessel cells

**Figure 1:**
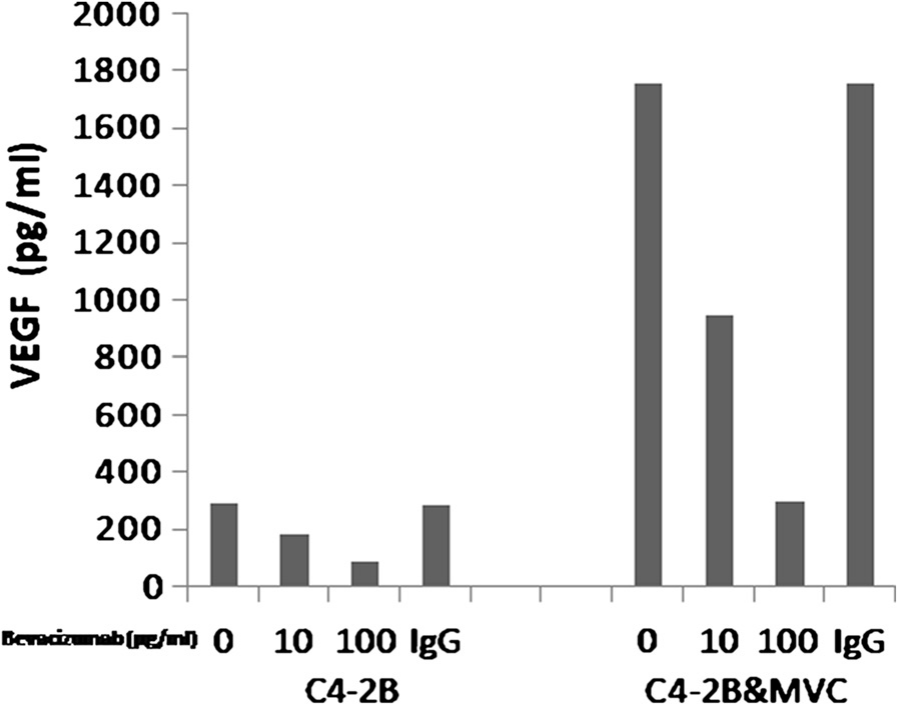
** VEGF expression after in vitro treatment with bevacizumab.** Both 10 and 100 μg/mL bevacizumab decreased the level of VEGF in C4-2B only, compared with control IgG. There were significant differences in the VEGF levels between the 10 or 100ug/ml bevacizumab and control IgG (P < 0.01). Human bone metastatic prostate cancer cell co-cultured with human microvessel cell expressed 6 times more VEGF than did tumor cultured cell only, and this level significantly decreased after treatment with 10 or 100 μg/mL bevacizumab.

To determine the concentration of bevacizumab needed for neutralizing the secreted VEGF by bone metastatic prostate cancer C4-2B cell line, ELISAs were performed to measure the levels of VEGF in conditioned media in C4-2B and C4-2B co-cultured with microvessel cells under bevacizumab or control IgG treatment. The level of VEGF from cells with bevacizumab or control IgG treatment is shown in Figure 1. The level of VEGF secreted by human bone metastatic prostate cancer C4-2B cell line co-cultured with microvessel cells was much greater than that secreted by C4-2B only. Both 10 and 100 μg/ml bevacizumab decreased the level of VEGF secreted by C4-2B, compared with control IgG. There were significant differences in the VEGF levels between the 10 or 100 ug/ml bevacizumab and control IgG (P < 0.01). Treatment with 100 μg/ml bevacizumab caused a more pronounced decreased in VEGF than treatment with 10 μg/ml bevacizumab. The level of VEGF was significantly increased when tumor cells were co-cultured with vascular endothelium. The levels of VEGF in co-culture media were 5.97 times greater than that in medium from C4-2B only. VEGF was reduced in C4-2B to 187.53 ± 23.79 pg/mlafter treatment with 10 μg/ml bevacizumab and 91.06 ± 19.82 pg/ml after treatment with 100 μg/ml bevacizumab, and in C4-2B co-cultured with microvessel cell VEGF was reduced to 949.42 ± 177.88 pg/ml after treatment with 10 μg/ml bevacizumab and 297.20 ± 69.27 pg/ml after treatment with 100 μg/ml bevacizumab,. There were significant differences in the VEGF levels between the 10 and 100 μg/ml bevacizumab treatment cells and control IgG treatment cells (P < 0.01, Figure 1). A high concentration of bevacizumab was more effective than a low concentration on reducing VEGF in C4-2B cells and C4-2B cells co-cultured with microvessel cells.

### Bevacizumab inhibited cell proliferation in C4-2B

**Figure 2:**
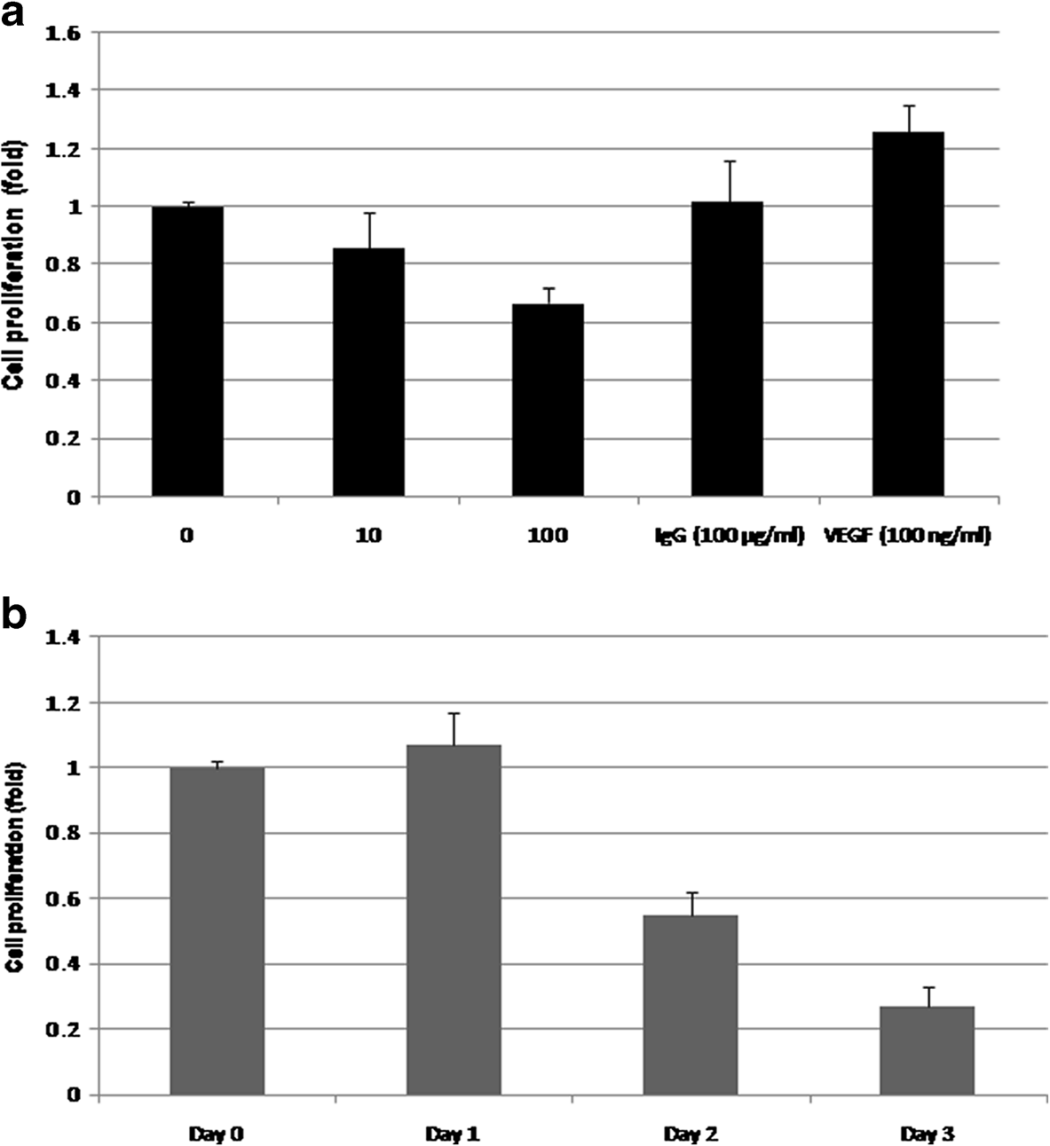
** Bevacizumab inhibits the growth of bone metastasis prostate cancer cell line C4-2B. a**. Different concentrations of bevacizumab inhibited the cell proliferation of C4-2B in a dose-dependent manner after 2-day incubation determined by mitochondrial MTS assay. Ig G (100 μg/ml) did not decrease the growth of C4-2B. cells. VEGF (100 ng/ml) enhanced the growth of C4-2B cells. **b**. The effect of bevacizumab on the inhibitory proliferation of C4-2B was gradually increased with a time-dependence. The relative fold was assigned as 1.0 in the absence of bevacizumab treatment. **means P < 0.01, significant differences from the bevacizumab treated with untreated group.

Because the increased production of VEGF drives angiogenesis related to tumor progression, we investigate the possibility that neutralization of VEGF may interrupt by the growth of bone metastatic prostate cancer C4-2B cell line. When C4-2B cells were exposed to bevacizumab (0, 10, 100 μg/ml) for a 2-day incubation, the growth of C4-2B was inhibited in a concentration-dependent manner, whereas the control IgG did not affect the growth C4-2B cells, and VEGF enhanced the proliferation of C4-2B cells (Figure 2a). At day 3 bevacizumab (100 μg/ml) inhibited the proliferation of C4-2B cells by 83% (Figure 2b). These data suggest that bevacizumab significantly inhibited cell proliferation in bone metastatic prostate cancer cells.

### Bevacizumab suppressed of angiogenesis in vitro

**Figure 3:**
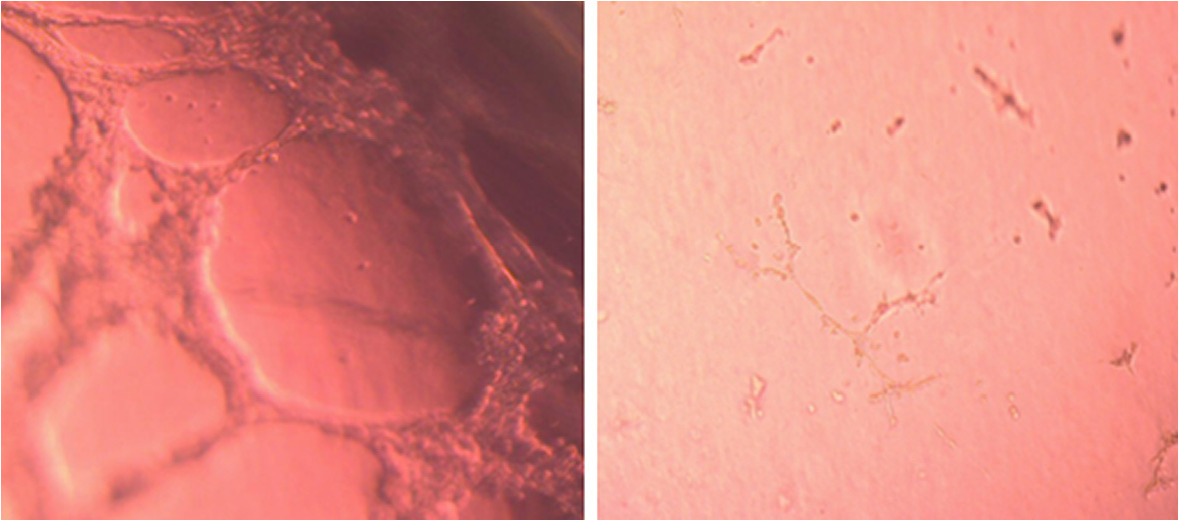
Suppressed tube formation of human microvessel by conditioned media from C4-2B cells treated with bevacizumab (right) or control IgG (left).

Based on the effect of different concentrations of bevacizumab on the proliferation in C4-2B cells, 100 μg/ml of bevacizumab would be used in the angiogenesis and invasion assay in vitro. Human microvessel cells developed complex meshlike structure patterns (grade 5) when grown in a growth-factor gel matrix. At 10 hour after control IgG treatment, the cells formed complex meshlike structure patterns (Figure 3, left). After treatment with bevacizumab (100 μg/ml), the cells showed a migration/alignment pattern (grade 1, Figure 3, right). The average total capillary tube length in human microvessel cells with IgG, or bevacizumab (100 μg/ml) was 1255.31 ±134.90 and 195.04 ± 26.67 μm, respectively (P < 0.01).

### Bevacizumab reduced C4-2B cell invasion

**Figure 4:**
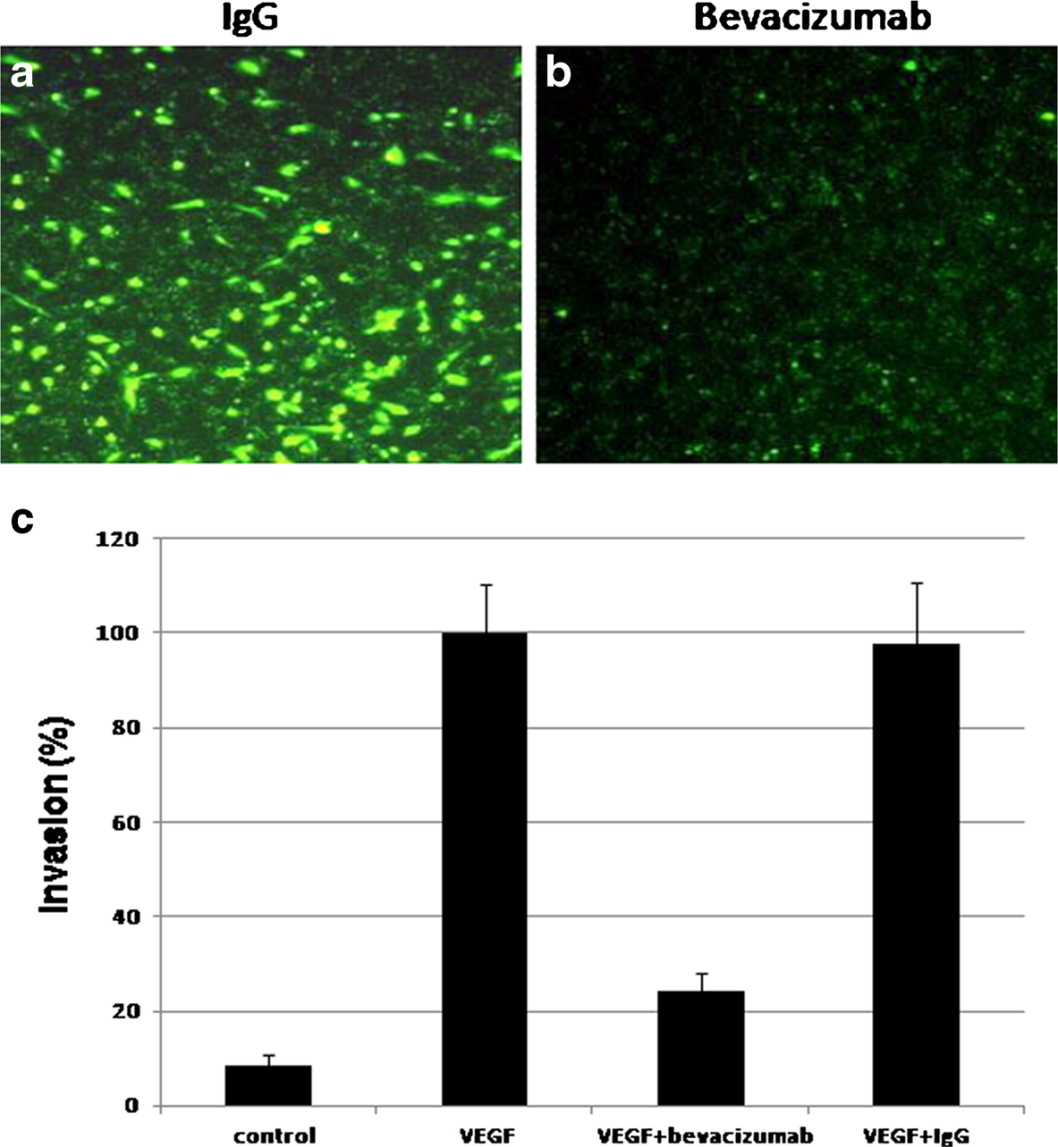
** Bevacizumab reduced the ability of invasion in C4-2B (b), comparing with an equal amount of IgG treatment (a)**. In the invasion assay, we seeded cells on the top of the Matrigel and added VEGF to the lower chamber. Invasive cells penetrate Matrigel and end up on the other side of the Matrigel. We estimated invasion by measuring the fluoresence intensity in the fluorescence microplate reader and counting the number of invading cells, and setting the average of invading cell numbers of C4-2B with VEGF added to the lower chamber as 100%. The results showed that VEGF-mediated invasion of C4-2B was suppressed by bevacizumab, and not by IgG1. (P < 0.01, Figure 4c).

The level of VEGF is known to correlate with prostate cancer invasion and metastasis in bone. We performed in vitro invasion assay to estimate whether bevacizumab reduced C4-2B cell invasion. RPMI-1640 without FBS was added to the lower chamber as a negative background control, RPMI-1640 with 5%FBS was added to the lower chamber and C4-2B cells without treatment were added to the upper chamber as a positive control. In order to express the direct role of VEGF on the invasion of C4-2B cells, the recombinant human VEGF as a chemoattractant was added to the lower chamber. VEGF induced C4-2B cells to invade through the Marigel. In the absence of VEGF, the invasion was very low. With 100 μg/ml of bevacizumab in the upper chamber, significantly less numbers of C4-2B cells migrated into the lower chamber, and IgG1 did not inhibit the invasion (Figure 4a and b). The result of the fluorescence microplate reader showed that the fluoresence intensity in the chamber with bevacizumab (100 μg/mL) was significantly lower than that in the chamber with control IgG1 (Figure 4c). Bevacizumab was high significantly decreased C4-2B cell invasion, comparing with control IgG (Figure 4, P < 0.01)

## Discussion

In solid tumor, such as prostate cancer, there is the chance that the cancer will become advanced and spread to the bone. In prostate cancer, the most common site of a recurrence is the bone. In fact, approximately 80% of prostate cancer recurrences are in the bone [6]. If the cancer metastasizes to distant sites, the 5-year survival rate is the only 31%. Importantly, once tumors metastasize to bone, they are virtually incurable and result in significant disease morbidity prior to a patient’s death. Bone metastases can lead to pain, pathological fractures, nerve compression syndromes, and hypercalcemia. Current treatments are mainly palliative. Despite the high incidence and serious consequences of skeletal metastasis of prostate cancer, the mechanism underlying this osteotropism is unclear. However, it is clear that VEGF has been implicated in various carcinogenesis and metastasis as well as in angiogenesis. VEGF is expressed by prostate cancer at a high level [7-9], and its expression correlates with increasing grade, vascularity, and tumorigenicity [9,10]. These relationships have been observed in human as well as in animal models of prostate cancer. High VEGF levels in prostate cancer are associated with poor prognosis. In addition, VEGF produced by tumor cells affects bone remodeling and might, therefore, facilitate nesting of metastatic cells in bone [11]. Bevacizumab is a recombinant, humanized monoclonal antibody that inhibits the binding of vascular endothelial growth factor (VEGF) to its receptors. Several experimental studies have examined the extent to which VEGF inhibitors or VEGF targeted agents prevent tumor cell growth and metastasis in vitro and in vivo [12-20]. In this study, we focus on the effect of bevacizumab on human bone metastatic LNCaP-derivative C4-2B prostate cancer cell line.

Angiogenesis is one of the critical events required in the cancer metastatic process. VEGF is a specific stimulator of vascular endothelial cell proliferation and tumor angiogenesis. VEGF is produced in response to various cellular and environmental stimuli. VEGF is overexpressed in many human neoplasms [4,5,7,9,20-22]. This expression is associated with increased tumor size, necrosis and tumor angiogensis. New blood vessels that grow within the tumor secondary to VEGF expression are structurally and functionally irregular, as they exhibit dead ends, disordered blood flow, and increased permeability. These irregularities in blood flow lead to further tumor hypoxia and subsequent increases in VEGF production [23,24]. In this study, we confirm that human bone metastatic prostate cancer cell line C4-2B has a higher level of VEGF than its parental cell line LNCaP, although both of cell lines have high levels of VEGF expression. We found that VEGF production significantly increased 6-fold when bone metastatic prostate cancer cells were cocultured with vascular endothelium.

VEGF exhibits the effects on the growth and progression of neoplasia. Several studies have shown a correlation between increased VEGF expression and tumor growth [16-23]. Recent studies have indicated that bevacizumab treatment results in a dose-dependent inhibition of tumor growth in vitro and in vivo [18,24,25]. In our study, bevacizumab gave a dose-dependent and time-dependent reduction of cell proliferation in human bone metastatic prostate cancer cells.

Metastasis is an extraordinarily complex process. To successfully colonize a secondary site a cancer cell must complete a sequential series of steps before it becomes a clinically detectable lesion. These steps typically include separation from the primary tumor, invasion through surrounding tissues and basement membranes, entry and survival in the circulation, and arrest in a distant target organ. These are usually, but not always, followed by extravasation into the surrounding tissue, survival in the foreign microenvironment, proliferation, and induction of angiogenesis. The treatment against any steps may affect the formation of metastasis. Our results show bevacizumab significantly decreases the ability of invasion and angiogenesis formation in human bone metastatic prostate cancer cells.

## Conclusions

In conclusion, anti-VEGF therapy has an inhibitory effect on human bone metastatic prostate cancer cells. Neutralization of VEGF disturbs the multistep process of metastasis including proliferation, angiogenesis and invasion. Anti-VEGF therapy is a potential adjuvant treatment strategy for the treatment of human bone metastatic cancer.

## Abbreviations

VEGF, Vascular endothelial growth factor; bFGF, Basic fibroblast growth factor; IL-8, Interleukin 8; ELISA, Enzyme-linked immunosorbent assay.

## Competing interests

All authors declare there are no competing interests.

## Authors’ contributions

LY and SY carried out the experiments. LY, SY, CZ and YC participated in study design and statistical analysis. LY, KV and YC drafted the manuscript. All authors read and approved the final manuscript.
